# Recombinant *Lactococcus lactis* NZ9000 secretes a bioactive kisspeptin that inhibits proliferation and migration of human colon carcinoma HT-29 cells

**DOI:** 10.1186/s12934-016-0506-7

**Published:** 2016-06-10

**Authors:** Bo Zhang, Angdi Li, Fanglei Zuo, Rui Yu, Zhu Zeng, Huiqin Ma, Shangwu Chen

**Affiliations:** Beijing Advanced Innovation Center for Food Nutrition and Human Health, College of Food Science and Nutritional Engineering, China Agricultural University, No. 17 Qinghua East Road, Haidian District, Beijing, 100083 People’s Republic of China; Key Laboratory of Functional Dairy, Department of Food Science and Engineering, College of Food Science and Nutritional Engineering, China Agricultural University, Beijing, 100083 People’s Republic of China; College of Horticulture, China Agricultural University, Beijing, 100193 People’s Republic of China

**Keywords:** *Lactococcus lactis*, KiSS1, Functional protein, Delivery vector

## Abstract

**Background:**

Proteinaceous bioactive substances and pharmaceuticals are most conveniently administered orally. However, the facing problems are the side effects of proteolytic degradation and denaturation in the gastrointestinal tract. In recent years, lactic acid bacteria (LAB) have been verified to be a promising delivery vector for susceptible functional proteins and drugs. KiSS-1 peptide, a cancer suppressor, plays a critical role in inhibiting cancer metastasis and its activity has been confirmed by direct administration. However, whether this peptide can be functionally expressed in LAB and exert activity on cancer cells, thus providing a potential alternative administration manner in the future, has not been demonstrated.

**Results:**

A recombinant *Lactococcus lactis* strain NZ9000-401-kiss1 harboring a plasmid containing the gene of the tumor metastasis-inhibiting peptide KiSS1 was constructed. After optimization of the nisin induction conditions, the recombinant strain efficiently secreted KiSS1 with a maximum detectable amount of 27.9 μg/ml in Dulbecco’s Modified Eagle medium. The 3-[4,5-dimethylthiazol-2-yl]-2,5 diphenyl tetrazolium bromide and would healing assays, respectively, indicated that the secreted KiSS1 peptide remarkably inhibited HT-29 cell proliferation and migration. Furthermore, the expressed KiSS1 was shown to induce HT-29 cell morphological changes, apoptosis and reduce the expression of matrix metalloproteinase 9 (MMP-9) at both mRNA and protein levels.

**Conclusions:**

A recombinant *L. lactis* NZ9000-401-kiss1 successfully expressing the human *kiss1* was constructed. The secreted KiSS1 peptide inhibited human HT-29 cells’ proliferation and migration probably by invoking, or mediating the cell-apoptosis pathway and by down regulating MMP-9 expression, respectively. Our results suggest that *L. lactis* is an ideal cell factory for secretory expression of tumor metastasis-inhibiting peptide KiSS1, and the KiSS1-producing *L. lactis* strain may serve as a new tool for cancer therapy in the future.

## Background

Oral administration employing delivery systems like hydrogels, nanoparticles, microspheres, and lipid-based systems is a convenient way of delivering drugs. However, this strategy usually has poor efficiency for bioactive macromolecules such as peptides and proteins due to the harsh gastrointestinal environment, with proteolysis resulting in denaturation and degradation of susceptible components [1]. Strategies for the delivery of proteinaceous agents have been intensively explored [2–4] to reduce the burden of repeat dosages and increased cost to improve the therapeutic effect. Potential alternative route might be using living microorganisms or other improved physically coated delivery vectors [4, 5].

Lactic acid bacteria (LAB) are generally regarded as safe for human health. In the last two decades, the feasibility of using LAB as functional protein delivery vectors has been widely investigated [6]. *Lactococcus lactis*, a LAB model species, has been demonstrated to be a promising candidate for the delivery of functional proteins, mainly because this bacterium is a noninvasive and nonpathogenic organism with fewer secretary proteins and extracellular proteases produced [7]. Many different expression systems of *L*. *lactis* have been developed and used for heterologous protein expression [8–10]. The most commonly used system is the nisin-controlled gene expression (NICE) system, containing the nisin promoter [8]. Some successful examples using *L. lactis* as a functional protein-delivery vector have been reported [10–12]. In particular, use of an interleukin-10-secreting *L. lactis* to treat Crohn’s disease has passed phase I clinical trials [13], indicating that *L. lactis* is indeed a safe mucosal vector for functional protein delivery. Tumor-targeted reagents, anticancer drugs, and non-invasive insulin- and vaccine-delivery systems can be developed based on live *L. lactis* [14, 15].

Nowadays, in cancer research, more attentions have been paid on the blockade of the metastatic process at the early stage [16]. Therefore, there is growing interest in identifying metastasis suppressor genes, which may be involved in the anti-metastatic activity. Kisspeptins (KiSS1) are peptide products of the *kiss1* gene, which was first identified by Lee et al. [17] in 1996 as a suppressor of tumor metastasis in human malignant melanoma cells. Gene *kiss1* is predicted to encode a 145-amino-acid peptide that can be cleaved into smaller peptides, known as kisspeptin-54 (KP54), KP14, KP13, and KP10. KiSS1 has been studied in various types of cancer and has been suggested to play multiple roles in cancer development and in suppression of metastasis, such as thyroid cancer, oesophageal carcinoma, urinary bladder cancer, gastric carcinoma, epithelial ovarian cancer, and colorectal cancer [18–23]. These studies provided numerous experimental and clinical evidences that KiSS1 could be a potential molecular target for treatment of metastasis during cancer progression.

Some of previous studies used synthetic or purified KiSS1 form tissue to investigate the anti-tumor effect of KiSS1 on cancer cells [24–27], while others applied transfection method (using expression vector containing *kiss1* gene) to introduce KiSS1 into the tumor cells [28]. However, limited information is available with respect to the development of new strategies for delivering this proteinaceous anti-tumor molecule for therapeutic purpose in the future.

In this study, we evaluated whether *L. lactis* can be used as a cell factory to produce bioactive KiSS1 peptide and whether the secreted product has anti-tumor activity using the human colon cancer HT-29 as a model for in vitro experiments. Our results provide foundations for future exploitation of recombinant LAB for in vivo delivery of the proteinaceous agent KiSS1 in cancer therapy.

## Results

### Cloning and expression of *kiss1* in *L. lactis* NZ9000

**Fig. 1:**
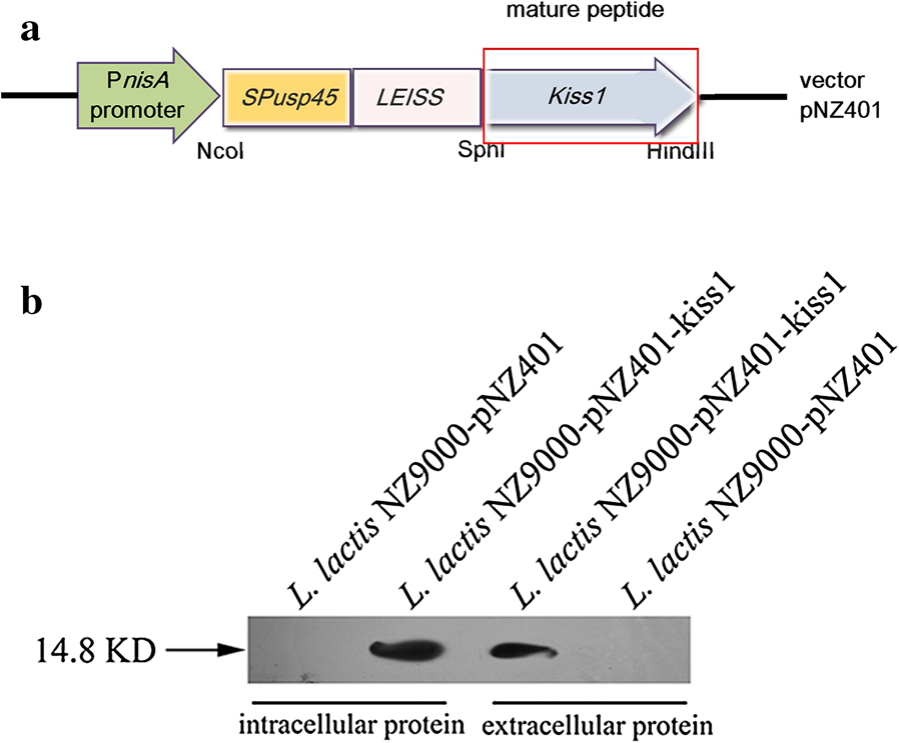
The expression of KiSS1 in *L. lactis* NZ9000. **a** Schematic representation of expression cassettes for controlled and targeted KiSS1 production in *L. lactis* NZ9000. **b** KiSS1 expressed in *L. lactis* NZ9000 as detected by western blot. *SP*_*USP45*_ DNA sequence encoding the Usp45 signal peptide, *LEISS* DNA sequence encoding the LEISSTCDA synthetic propeptide

The whole *kiss1* gene in length of 417 bp was amplified from the cDNA of human placenta by PCR and cloned into the nisin-induction vector pNZ401 which contained the Usp45 signal peptide and LEISSTCDA [29] synthetic propeptide (Fig. 1a). The resulting recombinant plasmids pNZ401 and pNZ401-kiss1 were transformed into *L. lactis* NZ9000, respectively. After induction with 10 ng/ml nisin for 1 h, the total cell protein and the secreted protein in the culture supernatant were separately extracted. Western blotting assay using anti-KiSS1 antibody revealed the expected 14.8-kD protein product in both the total protein extract and the extracellular protein extract from the recombinant strain harboring pNZ401-kiss1 (designated *L. lactis* NZ9000-pNZ401-kiss1) (Fig. 1b); however, no KiSS1 band was found in the parent strain (NZ9000) harboring the empty vector pNZ401 (designated *L. lactis* NZ9000-pNZ401), suggesting that *kiss1* was successfully expressed in *L. lactis* NZ9000.

### Optimization of expression conditions and detection of KiSS1 secretion

**Fig. 2:**
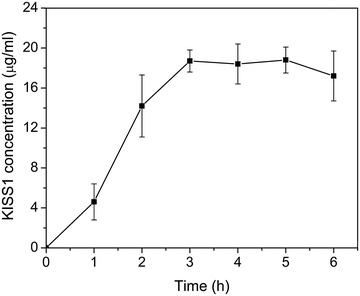
The concentration of KiSS1 under different induction time

To optimize the expression of the recombinant protein, different induction time (at the nisin concentration of 10 ng/ml) was tested. At each induction conditions, the amount of protein expressed was determined by indirect competitive enzyme-linked immunosorbent assay (ELISA). The results showed that KiSS1 production increased to a maximum detectable concentration of 18.7 μg/ml after 3–5 h induction (Fig. 2), and decreased thereafter. Thus the optimal condition for KiSS1 secretion was determined to be induction for 3 h with 10 ng/ml nisin.

**Fig. 3:**
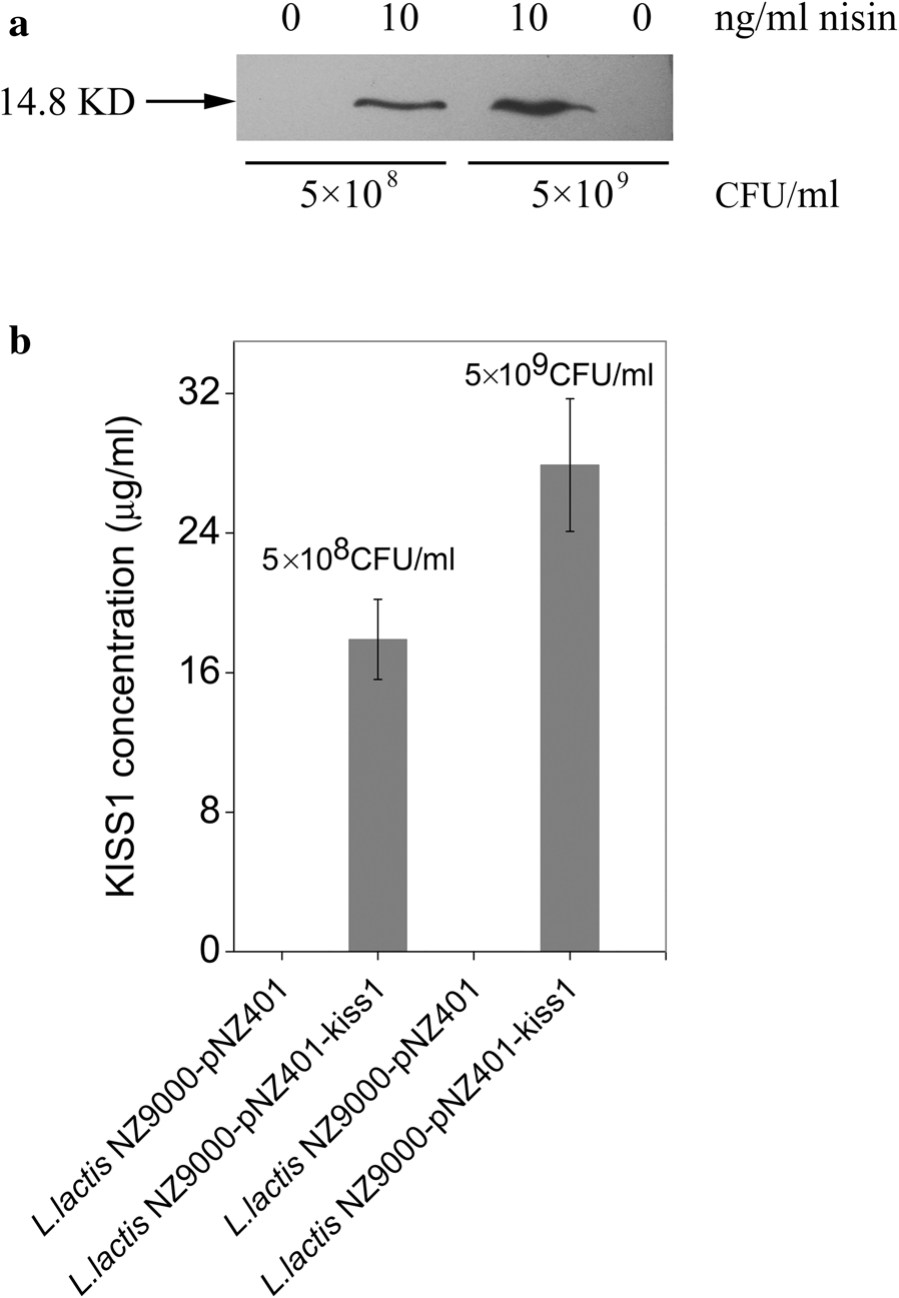
KiSS1 secreted by *L. lactis* NZ9000 in DMEM. **a** Detected by western blotting. **b** Detected by indirect competitive ELISA. Data are presented as mean ± SEM of three independent experiments

For the cell experiments, conditioned Dulbecco’s Modified Eagle medium (DMEM) was used to determine KiSS1 secretion. The recombinant strain *L. lactis* NZ9000-pNZ401-kiss1 as well as the control strain were cultured to an OD_600_ of 0.4, and then resuspended at different cell densities (5 × 10^8^ and 5 × 10^9^ CFU/ml) in DMEM before nisin addition. To determine the secretion of KiSS1, western blotting was performed to detect KiSS1 in the DMEM (Fig. 3a) and quantitative analysis was performed by indirect competitive ELISA (Fig. 3b). The results showed that the level of secreted KiSS1 was positively correlated with bacterial cell density; the maximal detected KiSS1 levels were 19.7 and 27.9 μg/ml in the *L. lactis* NZ9000-pNZ401-kiss1-conditioned DMEM at 5 × 10^8^ and 5 × 10^9^ CFU/ml cell densities, respectively. No corresponding products were found in the control strain *L. lactis* NZ9000-pNZ401 harboring empty vector. In addition, no detectable leaky expression of KiSS1 from the *L. lactis* NZ9000-pNZ401-kiss1 strain was observed (Fig. 3a), indicating that the P*nisA* promoter was highly efficient and fully functional for the induction of KiSS1 expression by nisin. Taken together, these results confirmed that KiSS1 is successfully heterogeneously expressed in the recombinant *L. lactis* NZ9000-pNZ401-kiss1 and effectively secreted into DMEM using 10 ng/ml nisin.

### *L.lactis* KiSS1 inhibits HT-29 cell proliferation and migration

**Fig. 4:**
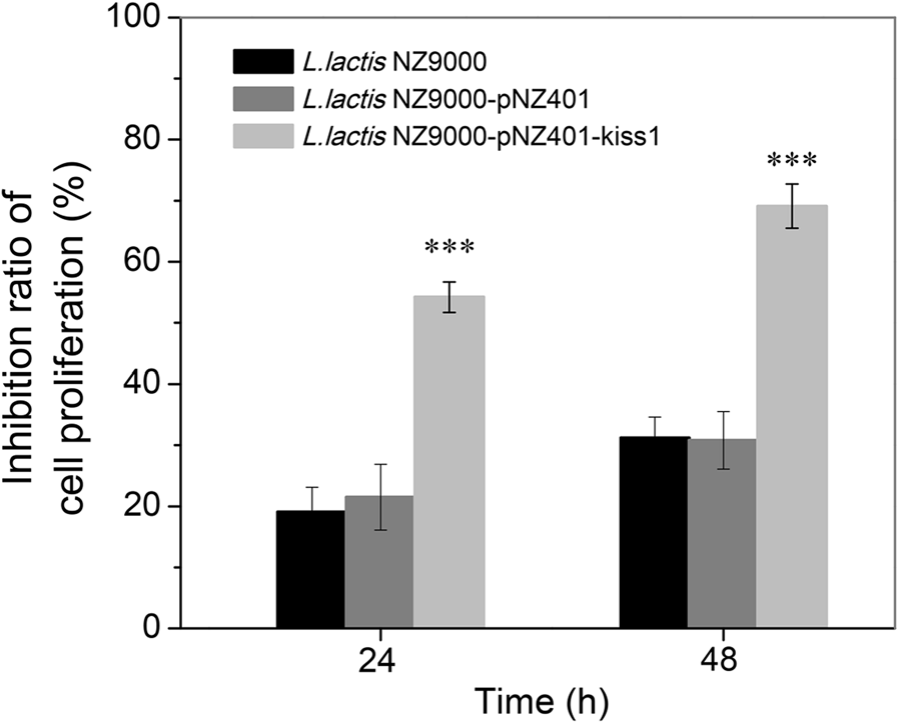
KiSS1 inhibits HT-29 cell proliferation. Data are presented as mean ± SEM of three independent experiments. *P ≤ 0.05, **P ≤ 0.01, ***P ≤ 0.001

**Fig. 5:**
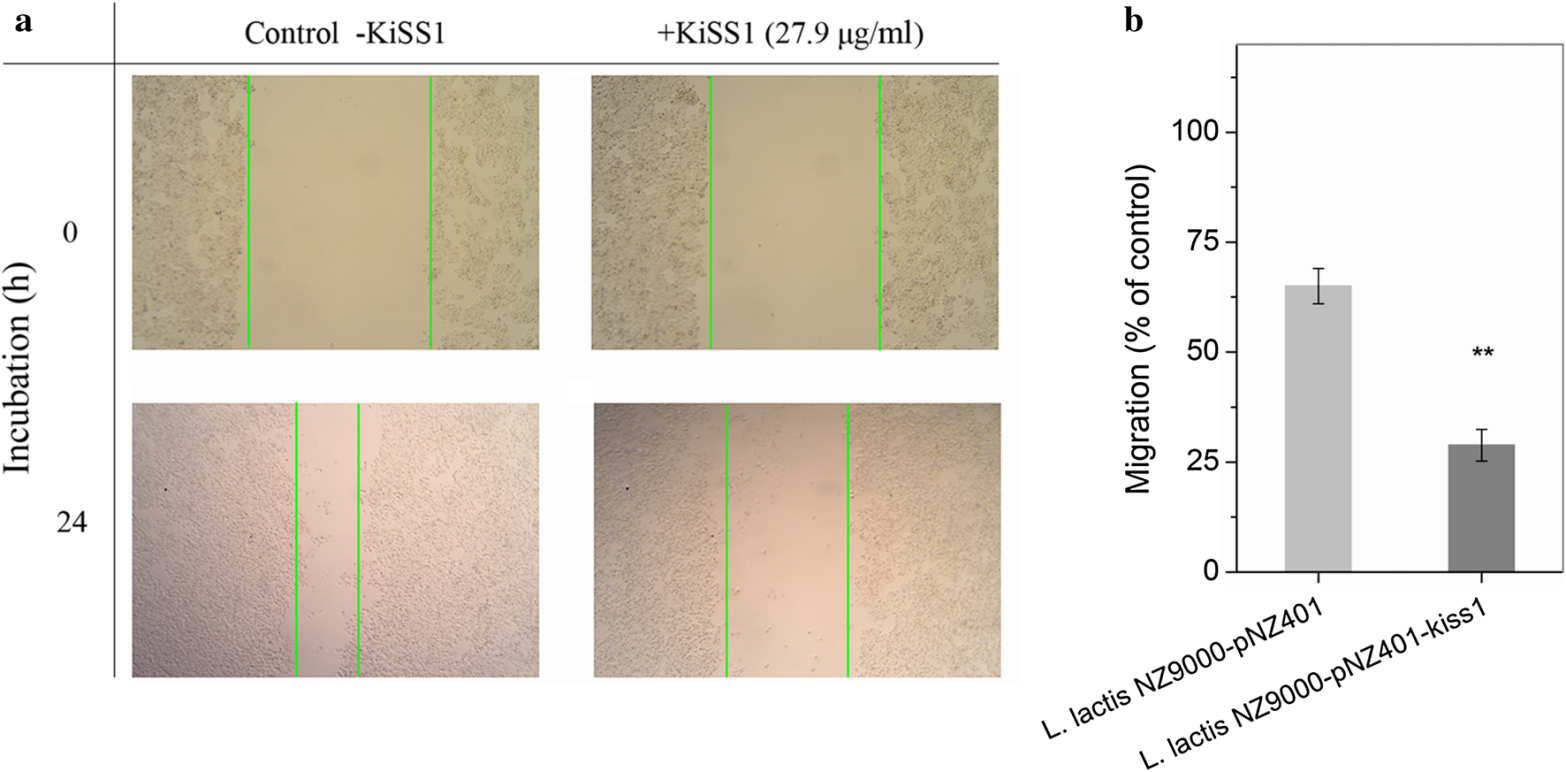
KiSS1 inhibits HT-29 cell migration. **a** Cell migration under *L. lactis* NZ9000-pNZ401 and *L. lactis* NZ9000-pNZ401-kiss1 treatment. **b** Cell migration ratio. Data are presented as mean ± SEM of three independent experiments. *P ≤ 0.05, **P ≤ 0.01, ***P ≤ 0.001. Magnification 100×

To evaluate whether the secreted KiSS1 was biologically active, we first tested the inhibition effect of KiSS1 on HT-29 cell proliferation using 3-[4,5-dimethylthiazol-2-yl]-2,5 diphenyl tetrazolium bromide (MTT) assay. The results showed that both the supernatant of DMEM conditioned with *L. lactis* NZ9000 and NZ9000-pNZ401 strains inhibited HT-29 cell proliferation; however, the rate of HT-29-growth inhibition by KiSS1 in recombinant *L. lactis* NZ9000-pNZ401-kiss1-conditioned DMEM was 2.52-fold (24 h) and 2.24-fold (48 h) higher (*P* ≤ 0.001) than that in *L. lactis* NZ9000-pNZ401-conditioned DMEM at those time points (Fig. 4). The total inhibition of HT-29 cell proliferation reached 54.2 % (24 h) and 69.1 % (48 h) by KiSS1. Thus the *L. lactis*-secreted KiSS1 was correctly processed, functionally folded and actively involved in inhibition of HT-29 cell proliferation. We further investigated the HT-29 cell migration in monolayer culture under different treatments (Fig. 5). HT-29 cells were firstly seeded on fibronectin-coated 6-well culture plates. After 24-h culture, cell monolayers were wounded and the culture medium was replaced by DMEM with or without KiSS1. After another 24-h culture, the results showed that, in the KiSS1-conditioned medium, the migration rate of HT-29 cells was only 44.5 % of that in KiSS1-negative medium (*P* ≤ 0.01), indicating that the *L. lactis*- secreted KiSS1 effectively inhibits HT-29 migration.

### KiSS1 induces cell morphological changes, apoptosis and a decrease of MMP-9 expression

**Fig. 6:**
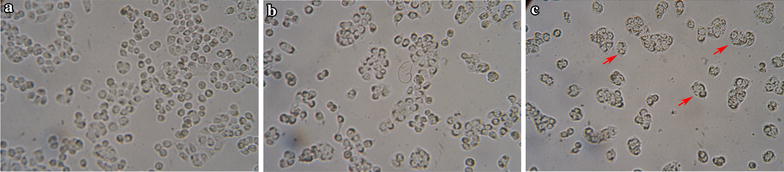
Photomicrographs of HT-29 cells treated with KiSS1 for 24 h. **a** Nontreated control. **b** Cells treated with *L. lactis* NZ9000-pNZ401. **c** Cells treated with *L. lactis* NZ9000-pNZ401-kiss1. *Red arrow* indicates apoptotic cell. Magnification 250×

The morphological changes of HT-29 cells cultured in *L. lactis* NZ9000-pNZ401-kiss1-conditioned DMEM displayed typical features of cell apoptosis/programmed cell death, i.e., various degrees of shrinkage, blebbing, aggregation and lack of obvious boundaries (Fig. 6), whereas cells growing in only DMEM or in *L. lactis* NZ9000-pNZ401-conditioned DMEM developed with clear, regular boundaries (Fig. 6a–c).

**Fig. 7:**
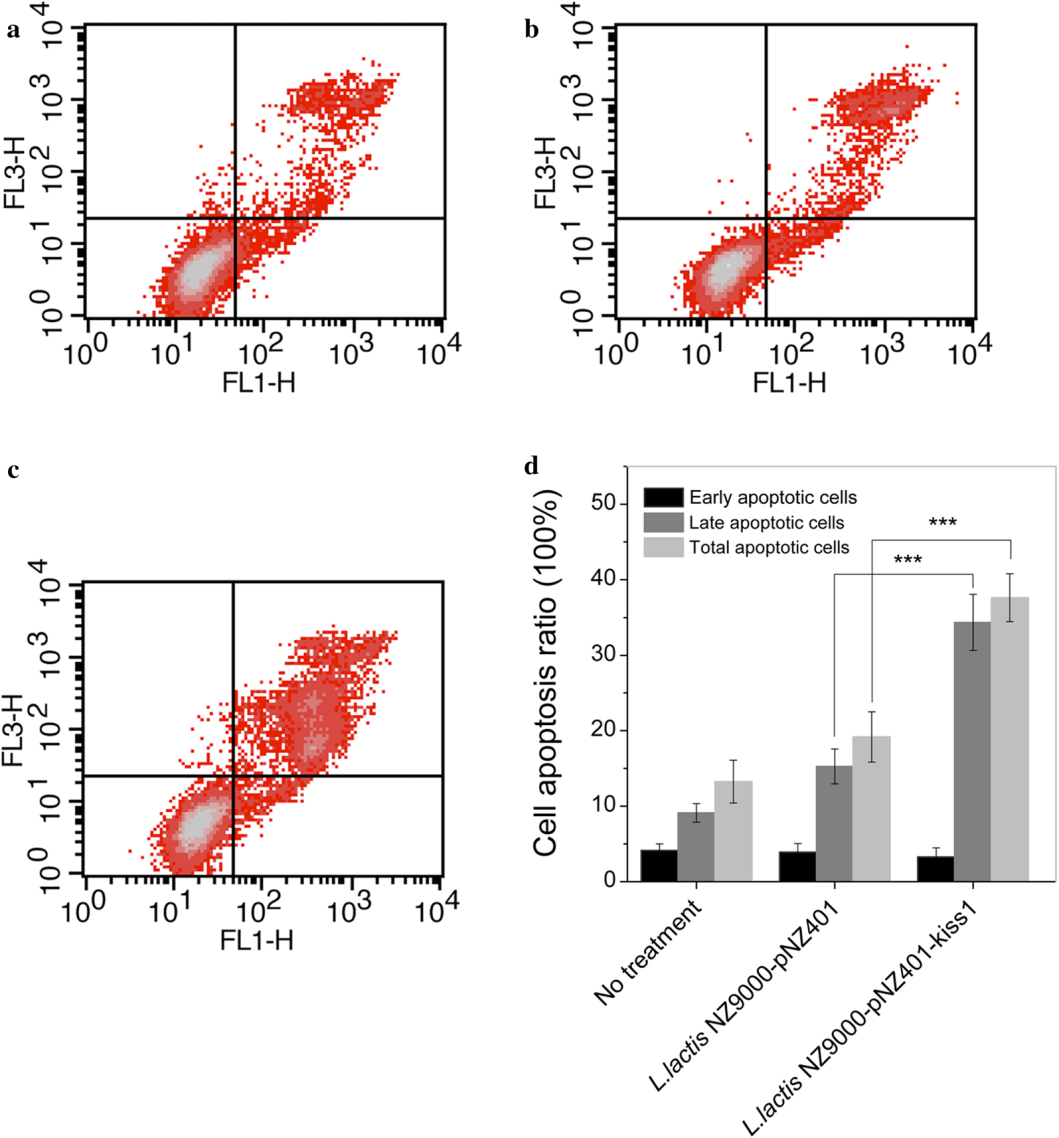
Effect of KiSS1 on HT-29 cell apoptosis. **a** Nontreated control. **b** Cells treated with *L. lactis* NZ9000-pNZ401. **c** Cells treated with *L. lactis* NZ9000-pNZ401-kiss1. **d** Percentage of apoptotic cells induced by KiSS1. *P ≤ 0.05, **P ≤ 0.01, ***P ≤ 0.001. Quadrant 2 (*upper right*) and quadrant 4 (*lower right*) in *panels* **a**, **b** and **c** indicate *late* and *early* apoptotic cells, respectively

To further confirm KiSS1 induction of cell apoptosis, analytical flow cytometry with Annexin V-PI (propidium iodide) double staining was used to assay the changes in HT-29 cells (Fig. 7). The apoptosis-inducing effect was observed in HT-29 cells at different cell cycle stages, mostly at the late stage of the cell cycle. The KiSS1-negative controls *L. lactis* NZ9000 and *L. lactis* NZ9000-pNZ401 and the KiSS1-positive *L. lactis* NZ9000-pNZ401-kiss1 all induced constitutive apoptosis, at a rate of 13.26, 19.18 and 37.63 % of total cells, respectively (Fig. 7a–c). However, the apoptotic rate induced by the KiSS1-positive *L. lactis* NZ9000-pNZ401-kiss1 was 1.96-fold (*P* ≤ 0.001) and 2.8-fold (*P* ≤ 0.001) of that by *L. lactis* NZ9000-pNZ401 and *L. lactis* NZ9000, respectively. These results confirmed that KiSS1 secreted by *L. lactis* has apoptosis-inducing effects on the HT-29 cell. Therefore, we infer that the inhibition of HT-29 cell proliferation by KiSS1 from *L. lactis* is probably through invoking, or mediating the cell-apoptosis pathway.

**Fig. 8:**
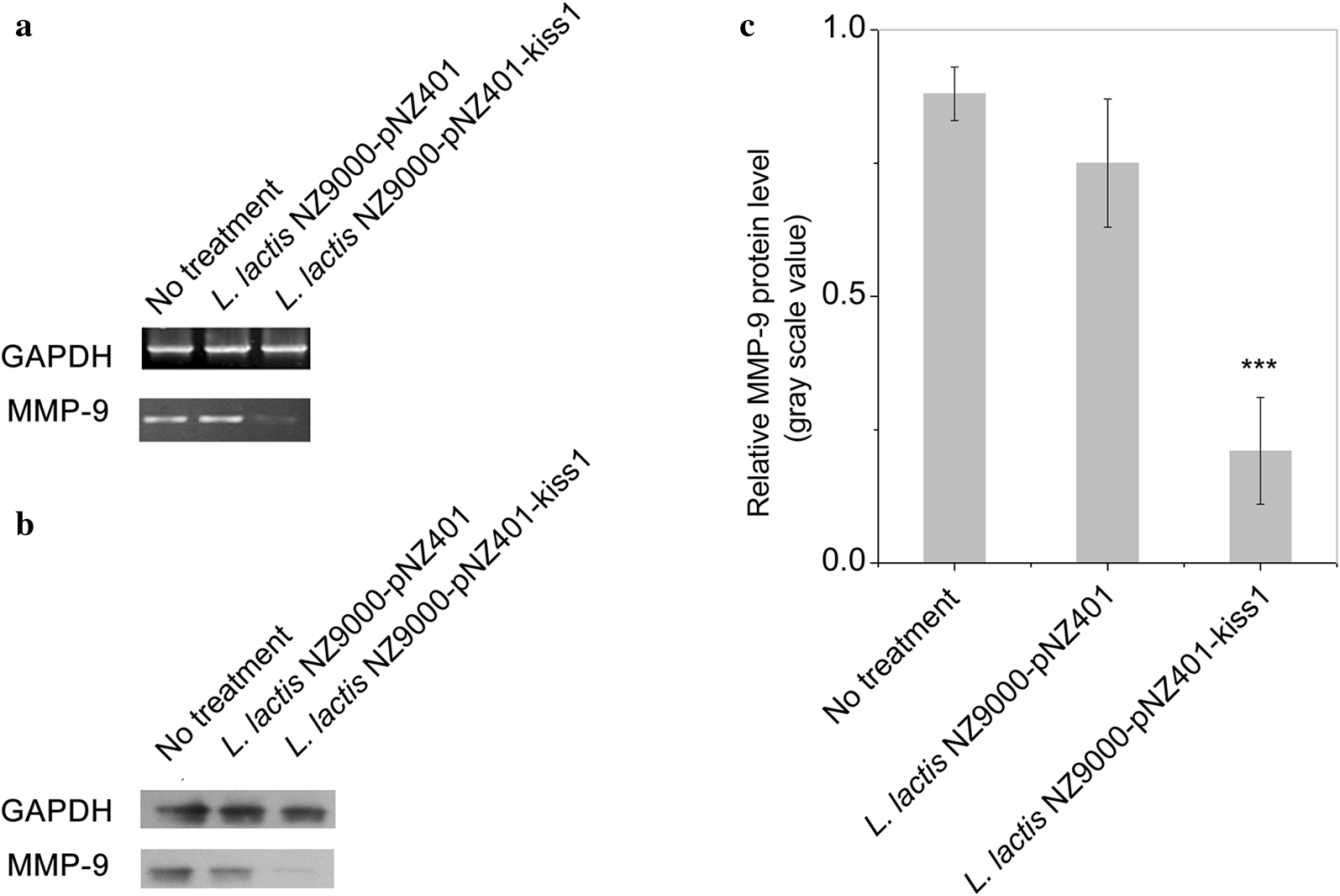
KiSS1 decreases MMP-9 transcription and expression. **a** MMP-9 transcription level. **b** MMP-9 protein level. **c** Relative MMP-9 protein expression level

Previous studies have shown that the expression level of matrix metalloproteinase 9 (MMP-9) is important for tumor cell invasion, migration and metastasis [29]. To examine whether KiSS1 affects MMP-9 expression, MMP-9 gene transcription and protein expression in HT-29 cells under the different treatments, with or without KiSS1-conditioned medium, were assayed (Fig. 8). Both the intensities of MMP-9 mRNA and protein bands in the cells treated with *L. lactis* NZ9000-secreted KiSS1 were lower than in the other two groups (no treatment and *L. lactis* NZ9000 harboring pNZ401 vector). As shown in Fig. 8c, the relative expression level of MMP-9 protein in the KiSS1 group decreased 3.75-fold compared to *L. lactis* NZ9000 harboring pNZ401 vector (*P* ≤ 0.001), suggesting that the expression of MMP-9 in HT-29 cells was downregulated by the *L. lactis* NZ9000-secreted KiSS1, thus exerting the inhibition effects on cell proliferation and migration.

### KiSS1 downregulates MMP-9 expression through G-protein coupled receptor 54 (GPR54) and activated ERK-MAPK signaling pathway

**Fig. 9:**
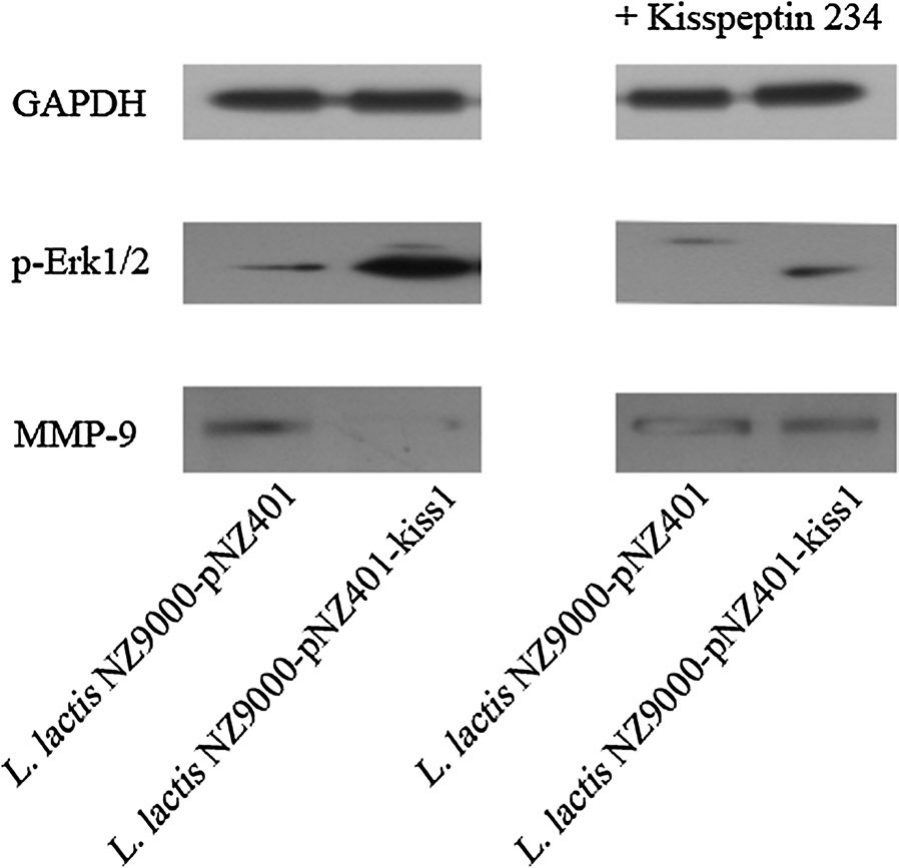
The expression of p-Erk1/2 and MMP-9 under different conditions (KiSS1 vs. KiSS1 plus GPR54 inhibitor). The expression of GAPDH was used as control in each condition. Kisspeptin 234, GPR54 inhibitor

It is reported that the downregulation of MMP-9 via GPR54 and ERK-MAPK signaling pathway was involved in the inhibitory effect of KiSS1 on the migration and invasion of cancer cells [30, 31]. To confirm this and to investigate the specificity of KiSS-1 action, kisspeptin 234 (GPR54 inhibitor), a 10-amino acid peptide antagonist for the kisspeptin-1/GPR-54 signaling system, was used in the inhibition experiments and the expression levels of Phosphor-Erk1/2 (ERK-MAPK pathway) and MMP-9 were evaluated. As shown in Fig. 9, KiSS1 expressed in *L. lactis* NZ9000-kiss1 obviously activated the expression of Phosphor-Erk1/2 and reduced the expression of MMP-9. On the contrary, when the inhibitor, kisspeptin 234, was added, the expression of Phosphor-Erk1/2 was significantly inhibited while the expression of MMP-9 was reversed. These results indicated that KiSS1 expressed in *L. lactis* effectively downregulated the expression of MMP-9 through the previously reported G-protein coupled receptor 54 and the activated ERK-MAPK signaling pathway.

## Discussion

The safety and efficacy of the food-grade microorganism *L. lactis* as oral vectors for mucosal delivery has been widely confirmed [6, 14]. Bioactive molecules delivered by this bacterium for therapeutic purpose (using both in vitro and in vivo models) includes anti-TNFα nanobodies for treatment of inflammatory bowel disease (IBD), glucagon like peptide-1 (GLP-1) for type 2 diabetes, leptin for body weight control, HPV-16 E7 protein for cervical cancer, catalase for IBD/colorectal cancer, among others [14]. In this study, we further verified that *L. lactis* was an excellent candidate as a delivery vector that can be engineered to secrete the anti-cancer peptide KiSS1 using the nisin-inducible expression system. In addition, it was demonstrated that tumor growth can be inhibited by the LAB strain itself [32, 33] due to the LAB’s ability to produce exopolysaccharides [34, 35], which is also an attractive feature for using *L. lactis* to deliver anti-cancer bioactive protein. We also observed that, as shown in Figs. 4 and 6, the control groups (*L. lactis* NZ9000 and *L. lactis* NZ9000-pNZ401) can inhibit HT-29 proliferation and induce cell apoptosis.

KiSS1 and its receptor KiSS1R have been studied for their metastasis-suppressing ability in numerous human cancer cells [20, 22, 24]. For example, it is found that high expression of KiSS1 can inhibit proliferation and invasion of tumor cells via lipofectin transfection of expression vector containing full-length KiSS1 complementary DNA (cDNA) into the tumor cell [28]. KP10, a short 10-amino acid peptide of KiSS1, can also inhibit proliferation and migration of tumor cells in vitro and in vivo [25, 36, 37]. We therefore hypothesized that expression of biologically active KiSS1 would inhibit proliferation and migration of different kinds of cancer cells. As an example shown here, *L. lactis* NZ9000 was demonstrated to be a cell factory for the secretion of biologically active KiSS1 protein, exerting inhibition effects on human colorectal cancer HT-29 cells.

Matrix metalloproteinases (MMPs) play crucial roles in invasion, metastasis and regulation of the signaling pathways controlling tumor cell growth, survival, invasion, inflammation and angiogenesis [38–40]. MMP-9 can release factors sequestered in the extracellular matrix, including VEGF, TGF-β and FGF-2, and stimulate proliferation and migration of endothelial cells [38, 41]. It is evidenced that KiSS-1 can suppress MMP-9 expression [42]; we therefore tested whether our expressed protein had such activity. The results firmly confirmed that KiSS1 secreted from our recombinant *L. lactis* NZ9000-pNZ401-KiSS1 strain effectively downregulated the expression of MMP-9. The reason for this is that KiSS1 is known to activate the MAPK pathway via GPR54 signaling, suppressing NFκB binding to the *MMP*-*9* promoter and thus downregulating MMP-9 expression [31]. This, in turn, reduces the survival rate, inhibits metastasis and induces dormancy of cancer cells. Here, by using GPR54 inhibitor, we further confirmed that KiSS1 expressed in *L. lactis* can effectively downregulate the expression of MMP-9 via GPR54 and the ERK-MAPK signaling pathway. These results were in accordance with previous findings for KiSS1 [43–45], warranting further exploration of the effectiveness of KiSS1-secreting *L. lactis* in vivo at inducing apoptosis, and inhibiting cell proliferation and metastasis.

## Conclusions

The human tumor-metastasis inhibitor protein KiSS1 was successfully expressed in *L. lactis*. The secreted KiSS1 remarkably inhibited human colonic epithelial HT-29 cell proliferation and migration mainly by inducing apoptosis and inhibiting MMP-9 expression, respectively. To our knowledge, this is the first time that the biologically active KiSS1 protein was successfully expressed in *L. lactis* and the anti-cancer effects of the secreted protein were evaluated. Our study suggests that *L. lactis* is an ideal vector for the secretion expression of anti-cancer agent KiSS1, which maybe a promising strategy for cancer therapy in the future.

## Methods

### Bacterial strains and growth conditions

The strains and plasmids used in this study are listed in Table 1. *L. lactis* NZ9000 was cultured in M17 (Difco) containing 0.5 % (w/v) glucose at 30 °C without shaking. To culture recombinant *L. lactis* strains, erythromycin was added at 5 μg/ml. *Escherichia coli* was incubated in LB medium at 37 °C with shaking at 220 rpm for 16 h.

**Table 1:** Bacterial strains and plasmids used in this study

Strains/plasmids	Relevant characteristics	Source or references
*Lactococcus lactis* NZ9000	MG1363 *pepN*::nisRK	[46]
*L. lactis* NZ9000-pNZ401	*L. lactis* NZ9000 harboring empty vector pNZ401	This study
*L. lactis* NZ9000-pNZ401-kiss1	*L. lactis* NZ9000 harboring pNZ401-kiss1	This study
*Escherichia coli* DH5α	Cloning host	Takara
*E. coli* DH5α-pNZ401	*E. coli* DH5α harboring empty vector pNZ401	Laboratory stock
*E. coli* DH5α-pNZ401-kiss1	*E. coli* DH5α harboring pNZ401- kiss1	This study
pNZ401	Em^r^, inducible expression vector containing the *nis*A promoter, derivative of pNZ8048	Laboratory stock
pNZ401-kiss1	pNZ401 carrying *kiss1* gene	This study

### Gene cloning, plasmid construction, and heterologous expression and secretion of KiSS1 by *L. lactis* NZ9000

The primers used in this study are listed in Table 2. Plasmid preparation, restriction endonuclease digestion, DNA ligation and other recombinant DNA techniques were performed according to standard methods [47]. The PCR product of *Homo sapiens* KiSS-1 metastasis-suppressor cDNA (GenBank accession number BC022819.1) was cloned into plasmid pNZ401, resulting pNZ401-kiss1. Plasmids pNZ401-kiss1 and empty control pNZ401 extracted from *E. coli* DH5α were both transformed into *L. lactis* NZ9000. For inducible expression, the recombinant *L. lactis* NZ9000-pNZ401-kiss1 and control (NZ9000-pNZ401) transformants were grown overnight in glucose-M17 (GM17) medium containing 5 μg/ml erythromycin. A 2 % (v/v) inoculum of the *L. lactis* strains was transferred to fresh GM17 broth and grown at 30 °C without shaking to an optical density at 600 nm (OD_600_) of 0.4–0.6, and then 10 ng/ml nisin (50 IU/ml, Sigma) was added and the culture was incubated for 3 h. To prepare conditioned medium for cell experiments, DMEM was used for KiSS1 secretion (DMEM-KiSS1 medium). The inoculated NZ9000-pNZ401-KiSS1 and NZ9000-pNZ401 strains were grown to OD_600_ of 0.4–0.6, harvested and washed twice with PBS (pH 7.2) buffer, then suspended in DMEM without fetal calf serum or antibiotics. Nisin (10 ng/ml) was added and the mixture was incubated for 3 h. The mixture was centrifuged and the supernatant was collected, neutralized with sodium hydroxide and filtered through a 0.22-μm membrane as DMEM-KiSS1 conditioned media. Fetal calf serum (10 % v/v) was added for incubation with HT-29 cells.

**Table 2:** Primers used in this study

Primer	Sequence (5′ → 3′)^a^	Description and location
Primer 1	ATCCATGGGTATGAAAAAAAAGATTAT	Upstream of SPP2-F, *Nco*I^b^
Primer 2	CATGCATGCTGCGTCACAAGTCGACGATA	Downstream of SPP2-R, *Sph*I
Primer 3	CATGCATGCATGAACTCACTGGTTTCTT	Upstream of *kiss1*-F, *Sph*I^c^
Primer 4	CCCAAGCTTCAGCCCCGCCCAGCGCTTC	Downstream of *kiss1*-R, *Hin*dIII
Primer 5	ACCACAGTCCATGCCATCAC	Upstream of GAPDH for RT-PCR
Primer 6	TCCACCACCCTGTTGCTGTA	Downstream of GAPDH for RT-PCR
Primer 7	GGT GGA CCG GAT GTT CCC	Upstream of MMP-9 for RT-PCR
Primer 8	GCC CAC CTC TCC TCC	Downstream of MMP-9 for RT-PCR

^a^The added restriction enzyme sites are underlined

^b^SPP2 includes Usp45 signal peptide and LEISSTCDA synthetic propeptide

^c^ *Homo sapiens* KiSS-1 metastasis suppressor: gb:BC022819.1 (cDNA clone MGC:39258 IMAGE:5443510)

### Western blot analysis and ELISA quantification of KiSS1

The induced recombinant *L. lactis* was harvested by centrifugation at 8000*g* for 10 min at 4 °C. Pelleted cells were washed three times in PBS, resuspended in PBS and disrupted by ultrasonicator. The supernatants were placed in fresh tubes and mixed with trichloroacetic acid at a ratio of 1:9 (v/v), and allowed to precipitate for 2 h at 4 °C. Then the mixture was centrifuged at 10,000*g* for 15 min at 4 °C. The liquid was discarded and the pellet was washed three times in ice-cold acetone. Finally, the pellets were air-dried and dissolved in PBS buffer. Each protein sample was mixed with 5× SDS loading buffer (250 mM Tris–HCl pH 6.8, 10 % w/v SDS, 0.5 % w/v bromophenol blue, 50 % v/v glycerol, 5 % w/v β -mercaptoethanol) and separated by 12 % SDS–PAGE, and then transferred to a nitrocellulose membrane using a semi-dry transmembrane system. Protein bands were detected using anti-KiSS1 rabbit monoclonal antibody (Santa Cruz Biotechnology). *L. lactis* NZ9000- pNZ401 (harboring empty vector pNZ401) was used as a control.

The concentration of the secreted KiSS1 was estimated by indirect competitive ELISA. Microtiter plates with 96 wells were coated with 100 μl antigen per well diluted in 50 mmol/l carbonate buffer (pH 9.8) to a concentration of 10 µg/ml and incubated overnight at 4 °C. Sample solutions and standard antigen (KiSS1 protein, Sigma) were respectively incubated overnight at 4 °C with an equivalent volume of anti-KiSS1 antibody, diluted 1:400 in 10 mmol/l PBS (pH 7.4) containing 10 g/l bovine serum albumin (BSA) and 1 g/l Tween 20 (PBS-BSA-T). The plates were washed four times the next day with 250 μl per well of 10 mmol/l PBS (pH 7.4) containing 0.5 g/l Tween 20 (PBS-T). After washing the plates, the wells were filled with 100 µl PBS-BSA-T per well to block residual free binding sites and incubated for 1 h at 37 °C. The plates were washed and then 100 µl per well of reactive mixtures of samples or standard antigen and antibodies were added and incubated for 1 h at 37 °C. After washing the plates, the wells were filled with 100 µl per well horseradish peroxidase-conjugated goat anti-rabbit IgG (Santa Cruz Biotechnology), diluted 1:20,000 in PBS-BSA-T. After incubation for 1 h at 37 °C, the plates were washed again and then 100 µl per well of 3,3′,5,5′-tetramethylenbenzidine (TMB, Amresco) substrate solution was immediately added. The plates were incubated for 15 min at 37 °C. Finally, 50 µl per well of 2 mol/l H_2_SO_4_ was added to stop the reaction. Absorption was determined at the dual wavelengths of 450 nm and 630 nm by Multiskan MK3 ELISA plate reader (Thermo Labsystems). PBS-BSA-T without anti-KiSS1 antibody was included as the blank, and PBS-BSA-T without sample was the control.

### HT-29 cell culture and assays

Human colonic epithelial HT-29 cells (ATCC HTB 38) were cultured in DMEM containing 10 % fetal calf serum, and 100 U/ml penicillin/streptomycin. Cell proliferation was analyzed by MTT assay. HT-29 cells were seeded in 96-well plates at a density of 10^5^ cells per well with DMEM containing 10 % fetal calf serum. After 24 h incubation, the medium was discarded and changed to DMEM-KiSS1 conditioned medium (100 μl per well). Cells were treated at 24 and 48 h. Medium was removed and then incubated with 100 μl MTT solution (0.5 mg/ml) for 4 h. The MTT is converted to blue formazan, which was then dissolved in 200 μl DMSO and the sample was read in a Multiskan MK3 plate reader (Thermo Labsystems). Absorption was determined at 540 nm. The MTT assay is based on the fact that the MTT can be converted into formazan crystals by viable cells, which determines mitochondrial activity. In cell populations, the total mitochondrial activity is related to the number of living cells. Therefore, MTT assay can be used to measure the in vitro inhibition effect of drugs on cell proliferation [48].

### Detection of apoptosis by flow cytometry

HT-29 cells were inoculated at 5 × 10^5^ cells per well in 6-well plates. After incubation for 12 h, the cells were attached to the plates. The medium was then discarded and replaced with DMEM-KiSS1 conditioned medium (2 ml per well) incubating for 24 h. HT-29 cells cultured in DMEM alone and in *L. lactis* NZ9000-401-conditioned DMEM were used as controls. Cells were then treated with Annexin V-PI (propidium iodide) cell apoptosis kit (Biyuntian Biotech Co. Ltd.). Apoptosis was detected by flow cytometry using a FACScan (Becton–Dickinson).

### Wound-healing assay

HT-29 cells were seeded on fibronectin (40 μg/ml)-coated 6-well culture plates at a density of 10^6^ per well. After 24 h culture, cell monolayers were wounded with a pipette tip and washed with PBS. The culture medium was discarded and replaced with DMEM containing 1 % fetal calf serum with or without KiSS1 (27.9 μg/ml, 1 ml per well). Cells were grown for 24 h, and those migrating toward the wound regions were imaged and counted. The detail experimental procedures were performed according to previously described protocol [49].

### HT-29 cell RNA and protein extraction

Total RNA from HT-29 cells was isolated using TRIzol Reagent (Invitrogen), and then treated with RNase-free DNase I (Takara). RNA concentration was determined by spectrophotometry at 260 nm. The absence of residual DNA in the total RNA digested by DNase I was confirmed by PCR. Reverse transcription was carried out with M-MLV Reverse Transcriptase (Promega) in a 20-μl reaction volume containing 150 ng of random primers and 1 mM dNTP mix (Tiangen Biotech Co. Ltd.) to obtain the cDNA for gene-expression assays with specific primers (Table 2).

Total protein was isolated from HT-29 cells using cell lysis buffer for western blots (Biyuntian Biotech). The cells were treated with cell lysis buffer for 30 min, and then transferred, using a cell scraper, into a 1.5-ml tube. The cells were disrupted by ultrasonication (9 s, two times), centrifuged, and the supernatants were subjected to western blotting.

### Detection of the expression of MMP-9 and phosphor-Erk (1/2)

To detect the expression of MMP-9 and phosphor-Erk (1/2), HT-29 cells were separately treated with the expressed KiSS1 (*L. lactis* NZ9000-401-kiss1) and the KiSS1 plus kisspeptin 234 (300 nM for 2 h). The kisspeptin 234 trifluoroacetate salt, a GPR54 inhibitor, was purchased from Santa Cruz Biotechnologies Inc., (Santa Cruz, CA, USA). Anti-MMP-9 XP rabbit monoclonal antibody (Cell Signaling Technology), phosphor-Erk (1/2) MAPK mouse monoclonal antibody and GAPDH mouse monoclonal antibody (Biyuntian Biotech) were used for detecting the expression of MMP-9, phosphor-Erk (1/2) and GAPDH (used as control), respectively, according to standard Western blot procedures.

### Statistical analysis

Statistical analyses were performed using unpaired two-tailed Student’s t test. *P* values less than 0.05 were considered to be statistically significant. Data are presented as mean ± SEM of three independent repeats in each experiment. Data were analyzed using the SPSS software (Release 17, SPSS Inc., Chicago, IL, USA).

## Abbreviations

LAB: lactic acid bacteria
DMEM: Dulbecco’s Modified Eagle medium
MTT: 3-[4,5-dimethylthiazol-2-yl]-2,5 diphenyltetrazolium bromide
MMP-9: matrix metalloproteinase 9
NICE system: nisin-controlled gene expression system
KP54: kisspeptin-54
ELISA: enzyme-linked immunosorbent assay
GPR54: G-protein coupled receptor 54
IBD: inflammatory bowel disease
GLP-1: glucagon like peptide-1
cDNA: complementary DNA
MMPs: matrix metalloproteinases
GM17: glucose-M17
BSA: bovine serum albumin
TMB: 3,3′,5,5′-tetramethylenbenzidine
